# A Rare and Difficult Diagnosis: A Case of Hepatoid Carcinoma of the Gallbladder

**DOI:** 10.7759/cureus.43901

**Published:** 2023-08-22

**Authors:** Joana A Cabrera, Renata Monteiro, Inês Pinho, José Presa Ramos, Margarida Mota

**Affiliations:** 1 Internal Medicine, Centro Hospitalar de Vila Nova de Gaia/Espinho, Vila Nova de Gaia, PRT; 2 Internal Medicine, Liver Unit, Centro Hospitalar de Trás-os-Montes e Alto Douro, Vila Real, PRT; 3 Internal Medicine, Hepatology Unit, Centro Hospitalar de Trás-os-Montes e Alto Douro, Vila Real, PRT; 4 Infectious Diseases, Centro Hospitalar de Vila Nova de Gaia/Espinho, Vila Nova de Gaia, PRT

**Keywords:** polyp, gallbladder, hepatoid carcinoma, metastasis, hepatocellular carcinoma

## Abstract

Extrahepatic metastasis of hepatocellular carcinoma is usually associated with extensive intrahepatic lesions. Hepatoid adenocarcinoma is a rare variant of extrahepatic malignant adenocarcinoma that exhibits remarkable histological and immunohistochemical similarity to hepatocellular carcinoma, which can result in an underestimated diagnosis.

This case report describes a patient with a newly found gallbladder polyp. Following cholecystectomy, the initial histological and immunohistochemical evaluation suggested a metastasis of hepatocellular carcinoma. However, after multiple scans, no primary intrahepatic lesion was found. A subsequent review of the gallbladder specimens showed negative staining for CK7 and CK19, leading to a diagnosis of hepatoid carcinoma of the gallbladder.

## Introduction

Liver cancer is a leading cause of death in many countries, and its impact varies widely across the world. Primary liver cancer, including hepatocellular carcinoma, was among the top three causes of oncological death in 46 countries and among the top five causes of cancer-related death in 90 countries worldwide. It is predicted that the number of cases and deaths will rise over the next 20 years as the world population grows [1].

Hepatocellular carcinoma is a primary liver tumor, commonly associated with advanced liver disease (usually in patients with advanced fibrosis in cirrhotic stages) [2]. There are multiple etiologies for chronic liver disease that can lead to liver cirrhosis, but in most cases, it is impossible to isolate just one predisposing factor. There often are multifactorial aggressions, and the risk of developing liver damage when exposed to such different factors is cumulative [2,3].

Currently, the main cause of liver cirrhosis identified in patients with hepatocellular carcinoma is excessive alcohol consumption [4]. However, it is predicted that in the next decade, the main cause of advanced liver fibrosis will result from metabolic syndrome, leading to fatty liver and nonalcoholic steatohepatitis, a consequence of the modern lifestyle with poor alimentary choices and lack of physical activities, which contribute to a greater number of obese and overweight patients [1,3-7]. The diagnosis of liver cirrhosis is usually late, due to the absence of early symptoms and the lack of surveillance of susceptible individuals, a changing trend in recent years, with precocious diagnosis reflecting on benefits in morbidity and mortality, especially in the surveillance of risk patients with cirrhosis [2].

The appearance of extrahepatic metastases from hepatocellular carcinoma is described in the literature in 14%-36.7% of patients [6]. Although rare, patients with hepatocellular carcinoma have radiologically evident extrahepatic disease at the initial presentation, and a greater number of them develop metastasis with more advanced stages. Extrahepatic metastasis is also usually correlated with extensive intrahepatic lesions, often with vascular invasion [6]. There is also a correlation between high serum alpha-fetoprotein levels and a higher probability of extrahepatic metastases from hepatocellular carcinoma. The main metastatic sites described in the literature are the lung, lymph nodes, bone, and adrenal glands [6].

Hepatoid adenocarcinoma is a rare variant of extrahepatic malignant adenocarcinoma, consisting of foci of both adenomatous and hepatocellular differentiation, which has a remarkable morphological, histological, immunohistochemistry, and functional similarity to hepatocellular carcinoma, which leads to an underestimation in diagnosis due to its rare incidence compared to hepatocellular carcinoma [5,7]. The histogenesis of this tumor may be due to hepatoid differentiation in adenocarcinoma as the tumor progresses, or it may be from the bipotential cells that differentiate into cells with glandular and hepatoid features [5].

Immunohistochemistry is typically required to establish the diagnosis of hepatoid adenocarcinoma. Regarding immunohistochemistry, the typical markers of hepatoid differentiation are hepatocyte paraffin-1 (HepPar-1), CD10, and arginase-1 [5,8].

We present this case of hepatoid carcinoma of the gallbladder for its rarity and difficulty in diagnosis, to alert to this infrequent oncological entity and avoid future delays in diagnosis and treatment.

## Case presentation

We present a case report of a 60-year-old male with a history of chronic hepatitis C, genotype 3a, known since 2005, acquired by intravenous drug use in the past. In 2016, the patient was treated for 12 weeks with sofosbuvir and velpatasvir plus ribavirin achieving sustained viral response (SVR). He also had a history of chronic alcohol abuse, abstinent since 2017, type 2 diabetes mellitus, and arterial hypertension, controlled under pharmacological therapy.

After achieving a hepatitis C cure, he maintained a semestral outpatient follow-up, with clinical, analytical, and imaging evaluations with abdominal ultrasound to assess liver fibrosis progression into cirrhosis. In the three years of follow-up, the patient remained asymptomatic, with no evidence of liver disease progression, with a Fibrosis Index Score-4 (FIB-4) score under 2.0.

In 2019, a routine six-month ultrasound revealed a de novo 7-mm gallbladder polyp, lobulated hepatomegaly, and heterogeneous parenchymal pattern. No other changes were detected in the liver, bile ducts, or other abdominal organs.

**Figure 1:**
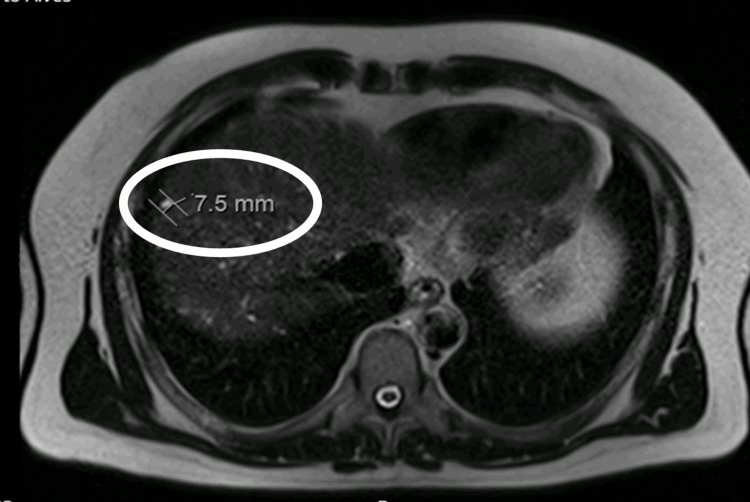
A 7.5-mm gallbladder polyp in the abdominal magnetic resonance.

The patient underwent an abdominal magnetic resonance that confirmed the gallbladder polyp, a pattern of diffuse hepatic steatosis, and several liver nodules suggestive of a malignant pattern, but not fulfilling the characteristics of hepatocellular carcinoma in the right lobe, the largest measuring 60 mm in the high hepatic convexity, with other measuring 46 mm in the V/VII segments and multiple scattered peri-centimetric nodules (Figure 1). These alterations coursed with an abrupt elevation of alpha-fetoprotein (42 ng/mL) (normal values: <8.5 ng/mL) and carcinoembryonic antigen (CEA) (5.1 ng/ml) (normal values: <2.5 ng/mL) levels.

A targeted liver biopsy (aiming for the largest right lobe nodule) was performed, displaying an exuberant ductular proliferation, with advanced fibrosis suggestive of cirrhosis. There was no evidence of cell atypia or necrosis in the tissues. The immunohistochemical evaluation tested anti-CK7, CD34, p53, glutamine synthase, and Glypican 3 antibodies, with no abnormal immunoreactions. Facing these results, the hypothesis of a liver malignancy became less probable; however, a definitive diagnosis could not be rendered.

The patient underwent a laparoscopic cholecystectomy because of de novo and rapidly growing gallbladder polyp (7-10 mm in about three months). The pathology of the surgical specimen showed a gallbladder with growth of exophytic structures from the internal mucosa suggestive of hepatocellular carcinoma, with a moderately differentiated pattern, without peripheral or vascular invasion. The most prominent data regarding the immunophenotype was the positivity of antibodies AE1AE3, CAM5.2, and Glypican.

Following the surgical approach, the patient was again scanned with an abdominal computed tomography (CT) and magnetic resonance to locate the primary lesion, and again, no abnormal or new structures were identified in comparison with previous examinations, describing stable hepatic nodules.

Faced with these findings described in the gallbladder suggestive of a secondary lesion, with features of hepatocellular carcinoma and with no other evidence of liver malignancy, a liver biopsy was repeated, again with no sign of liver cancer that would support the previous hypothetical diagnosis of gallbladder metastasis of hepatocellular carcinoma.

After the exhaustive and unsuccessful study, the multidisciplinary tumor board decided to review the gallbladder histology blades. The previous results were reconfirmed, but after an extended immunochemical panel, the results showed negativity for CK7 and positivity for CK19 (Table 1).

**Table 1:** Immunochemical panel of the gallbladder histology blades in both revisions. CEA: carcinoembryonic antigen

First revision	Second revision
AE1AE3 +	AE1AE3 +
CAM 5.2 +	CAM 5.2 +
Glypican 3 +	Glypican 3 +
CEA +	CEA +
Alpha-fetoprotein +	Alpha-fetoprotein +
	CK7 -
	CK19 -

After integrating the immunophenotypic pattern and the microscopic features, it was possible to identify the biliary lesion as a primary hepatoid adenocarcinoma of the gallbladder.

The patient remains under active clinical, imaging, and laboratory (including alpha-fetoprotein) surveillance to the present date, without needing other treatment or any other findings suggestive of malignancy.

## Discussion

Hepatoid adenocarcinoma has been reported to occur in the stomach, esophagus, duodenum, jejunum, colon, peritoneum, pancreas, lung, ovary, gallbladder, uterus, and other sites. Of these locations, the stomach is the most common site of hepatoid adenocarcinoma [5,9]. The hepatoid adenocarcinoma of the gallbladder remains a rare finding with little data available.

Hepatoid adenocarcinoma is a very unique variant that was first described by Ishikura in 1985. So far, less than 20 cases have been reported in the English literature, so it remains a less-known oncological entity, which might contribute to later diagnosis [9].

Hepatoid adenocarcinoma is a rare subtype of extrahepatic adenocarcinoma that closely mirrors both the morphology and functional attributes of hepatocellular carcinoma. Typically, the histopathological attributes of hepatoid adenocarcinoma consist of sizable or polygonal cells exhibiting abundant eosinophilic cytoplasm. It grows in either a solid or trabecular arrangement, occasionally demonstrating medullary proliferation. Moreover, within the lamina propria and/or submucosal regions, instances of papillary or tubular structures are frequently identified. The confirmation of bile production serves as evidence for the hepatoid nature of these cells [6,7].

In the initial stages of the diagnostic process, imaging examinations such as ultrasound, abdominal CT scans, or magnetic resonance play a pivotal role in comprehending the disease’s extent. However, these tests often fall short in distinguishing between hepatoid adenocarcinoma of the gallbladder and hepatocellular carcinoma. The frequent delay in diagnosing can be crucial in the local or distant spread of the disease, leading to worse outcomes in medical and surgical treatment, including delays in achieving optimal surgical timing [7-9].

These two oncological entities also share several clinical characteristics, such as elevated serum alpha-fetoprotein levels (although hepatoid adenocarcinoma of the gallbladder does not always produce alpha-fetoprotein). Other common characteristics include a higher prevalence in advanced age and aggressive tumor behavior (fast evolution with sudden severe symptoms) with poor prognosis [7,9,10].

The differentiation between hepatocellular carcinoma and hepatoid adenocarcinoma cannot be accomplished solely through morphological assessment; it necessitates discernment of variations in immunohistochemical patterns. Immunohistochemically, an array of liver-specific proteins, such as alpha-fetoprotein, albumin, transferrin, protein induced in the absence of vitamin K (PIVKA), and alpha-1-antitrypsin, has been detected within the cytoplasm of tumor cells both in hepatoid adenocarcinoma and hepatocellular carcinoma cases. On the other hand, the presence of the markers CK7 and CK19 is specific to hepatoid adenocarcinoma. It is imperative to include these markers in immunohistochemical analyses to effectively distinguish between these two entities [7-10].

Total surgical excision stands as the sole curative approach for neoplastic conditions affecting the gallbladder. However, achieving comprehensive resection frequently presents challenges due to the intricate proximity of essential anatomical structures neighboring the gallbladder and the fact that these malignancies have a notable tendency to infiltrate adjacent structures, adding to the complexity of complete removal [9,10].

With this practical example, we aim to remember the far less prevalent diagnostic hypothesis of hepatoid adenocarcinoma in elderly patients with high serum alpha-fetoprotein and multiple liver nodules, especially if they do not have underlying chronic liver disease. In this scenario, the diagnostic hurdles encompassed the uncommon tumor location, absence of evident symptoms during physical examination, and variation in the immune phenotype as revealed by immunohistochemical staining. It was necessary to integrate ample clinical data, imaging results, histological characteristics, and a range of immunohistochemical markers to arrive at the ultimate diagnosis. Early suspicion of this entity is crucial to guide immunohistochemical analysis to make a diagnosis as soon as possible, and awareness of its existence is very helpful in the differential diagnosis to avoid misdiagnosis as metastatic hepatocellular carcinoma. This is a malignant, aggressive disease that must be recognized as soon as possible to start suitable medical and/or surgical treatment [7,8].

## Conclusions

In our case, the patient had a known chronic liver condition, introducing additional confounding data to the evaluation of the case. Although rare, this entity deserves importance as a differential diagnosis between pathologists and to alert physicians to avoid possible misdiagnosis and inappropriate therapy.

We contend that this case merits attention due to its rarity, thus making its examination pertinent in order to raise awareness of this diagnostic possibility, prevent delays in diagnosis and treatment, and mitigate potential detrimental impacts on prognosis.
